# Clinical characteristics of ulcerative colitis in elderly patients

**DOI:** 10.1002/jgh3.12612

**Published:** 2021-07-12

**Authors:** Mingming Zhu, Zhihua Ran

**Affiliations:** ^1^ Division of Gastroenterology and Hepatology, Key Laboratory of Gastroenterology and Hepatology, Ministry of Health, Shanghai Inflammatory Bowel Disease Research Center, Renji Hospital, School of Medicine Shanghai Jiao Tong University, Shanghai Jiao Tong University; Shanghai Institute of Digestive Disease Shanghai China

**Keywords:** clinical characteristics, elderly, medical therapy, ulcerative colitis

## Abstract

The incidence of ulcerative colitis (UC) in elderly patients is increasing. Elderly UC patients are likely to exhibit distinct features both at diagnosis and during follow‐up. Age‐related problems, including complications, immune dysfunction, and multidrug use, make the diagnosis and treatment of elderly UC more challenging. Suboptimal treatment considering adverse events leads to poor clinical outcome in elderly UC patients. Here, we reviewed the epidemiology, clinical presentation, medical therapy, colorectal cancer surveillance of UC in elderly patients.

## Introduction

The incidence rate of ulcerative colitis (UC) has been reported to be increased across all age groups, including early childhood and elderly population.^1^, ^2^ Most Western countries define elderly UC patients as aged >65 years, including older‐onset UC patients (diagnosed at ≥65 years) and those transitioning to older age with their disease.^3^, ^4^

Compared with the younger population, the health status of the elderly patients is more heterogeneous, which makes the clinicians should assess an individual's frailty, rather than only considering an individual's chronological/biological age when making management decisions in the elderly. Suboptimal treatment considering the increased risk of infections and cancer development associated with the immunomodulators and biologics leads to poor clinical outcome in elderly UC patients.^5^

However, there are currently limited data on the characteristics, disease course, and treatment impact in elderly‐onset UC patients from clinical studies as elderly patients are often excluded from clinical trials. Therefore, the management decisions of UC in elderly patients are frequently based on expert opinion or consensus. In recent years, elderly patients with UC have attracted more and more attention from researchers. In this review, we discuss the clinical characteristics, medical therapy, and surveillance of UC in elderly patients.

## Epidemiology in elderly ulcerative colitis

There is a bimodal distribution phenomenon of UC onset, the first peak is seen in patients aged 30–40 years old, while the second one is seen in the elderly population and up to 15% of patients present after the of age 65.^6^ Although there are no published statistical data on the prevalence of UC in the elderly, most studies reported that the incidence rates of UC in older patients are increasing rapidly in the past few decades (from 1.1/100.000 to 19/100.000/year),^3^ including data from Asian countries.^7^, ^8^ In addition to the aging population, environmental factors may play more important roles in the incidence of UC in the elderly.^9^

## Clinical presentation in elderly ulcerative colitis

Studies showed that elderly UC patients were more likely to be male and less likely to have a family history of inflammatory bowel disease (IBD) or extraintestinal manifestations.^10^, ^11^ Similar to the symptoms in younger patients, older UC patients typically present abdominal pain, fecal urgency, and bloody diarrhea, and there are no significant changes in symptomatic severities between the two groups.^12^ Nevertheless, older‐onset UC patients are more likely to have left‐sided colitis than isolated proctitis and pancolitis.^13^, ^14^

Although previous studies have reported that older‐onset UC patients present similar surgery rate to that of non‐elderly patients,^15^, ^16^ including the topical review from European Crohn's and Colitis Organisation (ECCO).^12^ Other studies reported the converse, a recent meta‐analysis targeting only older‐onset UC reported a 1.36‐fold higher surgical rate.^17^ Recently, a large‐scale cohort study form Japan also reported that the surgery rate was significantly higher in older‐onset UC patients than that in non‐elderly patients.^18^

There are many reports that the hospitalization rate of elderly UC patients is significantly higher than that of non‐elderly UC patients.^12^, ^19^, ^20^ In an accompanying article by Moroi et al. (a Nationwide Study from Japan), the rate of in‐hospital death of elderly patients is higher.^21^ One of the reasons of worse outcomes is that older‐onset UC patients have less chances of receiving strong immunosuppressive therapies considering the comorbidities and adverse events such as infections.

## Medical therapy of elderly ulcerative colitis

As a special group, appropriate management and therapeutic strategies in the elderly UC patients need to be selected, balancing clinical outcomes and safety concerns. As well as younger individuals, treatment goals in older UC patients include induction and maintenance of disease remission. Generally, treatment choices are usually based on the location and severity of the underlying disease. However, when considering medical treatments in elderly UC patients, more attention should be paid to the interaction between their treatments and comorbid diseases, the susceptibility to infection and cancer risk.

### 5‐aminosalicylates

The 5‐aminosalicylates (5‐ASA) are the most commonly prescribed medications to induce and maintain remission for mild to moderate UC patients in elderly.^22^, ^23^, ^24^ In a population‐based study, 72% of the older‐onset UC patients were treated with 5‐ASAs.^14^ In the French prospective population‐based cohort (EPIMAD) study, the cumulative probability of receiving 5‐ASA therapy for older‐onset UC patients was 75%.^25^ Higher usages of 5‐ASAs in older UC patients are likely because of their perceived safety profile and potentially less aggressive disease course.

As well as oral mesalazine, topical drugs can also be used to treat mild to moderate UC in elderly, but it is worth to note that topical mesalazine may increase fecal incontinence rate of older patients.^26^

Overall, 5‐ASAs are considered safe and effective for the treatment of mild to moderate UC, but adverse events should be monitored closely in older patients, including some regular monitoring of renal function, interactions of 5‐ASA with other drugs, such as thiopurines, digoxin, warfarin, and isoniazid.

### Corticosteroids

Corticosteroids are proven to be effective in inducing remission in moderate to severe UC,^27^, ^28^ and it was reported that nearly a third of older‐onset patients received corticosteroids.^25^ However, long‐term use of corticosteroids can lead to osteoporosis, serious infections, and even death, especially in the elderly.^29^, ^30^, ^31^

Several large nationwide studies reported that corticosteroids exposure increased the risk of low bone mineral density significantly in UC patients.^32^ Compared with adult patients, elderly UC patients had higher risks of low bone mineral density and fragility fractures.

Another side effect of corticosteroids usage is the risk of infectious diseases. A population‐based cohort study reported that there was a correlation between the use of steroids within the previous 6 months and the development of serious infections in elderly‐onset UC patients.^29^ Generally, an age‐related decline in immune cell function and physical performance may contribute to the risk.

Furthermore, other adverse effects of corticosteroid usage are more frequent in elderly patients with UC, such as diabetes mellitus, worsening hypertension, cataracts, fluid retention, myopathy, glaucoma, sleep and mood disturbances.^32^ Therefore, we should pay more attention to avoid administering long‐term corticosteroids to elderly UC patients.

Budesonide MMX was designed for release throughout the colon, with high potency and low systemic corticosteroid activity.^33^ It has been proven to be more effective in inducing clinical remission in patients with mild to moderate UC but not for maintaining remission. Based on the present evidence, budesonide MMX 9 mg is a safe and effective alternative in inducing remission for elderly patients with UC.

### Thiopurine

Thiopurine (6‐mercaptopurine, azathioprine) has been proven to be effective and safe for maintaining remission in moderate to severe corticosteroid‐dependent disease in elderly UC patients,^12^, ^34^, ^35^ and its efficacy has been reported in Asian countries.^36^

Older‐onset UC patients may require prolonged thiopurine therapy (>12 months) for effectiveness. Sustained thiopurine treatment of more than 12 months in older‐onset UC patients was associated with a 70% reduction in risk for colectomy (hazard ratio [HR] 0.30; 95% confidence interval [CI] 0.15–0.58).^37^ However, the use of thiopurines has been shown to be associated with an increased risk for non‐Hodgkin's lymphoma, nonmelanoma skin cancer, urinary tract cancers in patients with UC, particularly with prolonged use and advancing age.^38^, ^39^, ^40^ In addition, the incidence of hepatotoxicity, opportunistic infection and tuberculosis increased significantly in elderly UC patients with thiopurines treatment.^37^

In fact, approximately 5–26% of older UC patients are prescribed thiopurines.^41^ Therefore, it is important to consider preventive strategies against adverse events while considering thiopurine therapy in elderly UC patients. NUDT15 gene variants should be routinely tested before initiating thiopurine therapy to avoid serious myelotoxicity from thiopurines.^42^ Avoiding prolonged use of thiopurines and combination with anti‐tumor necrosis factor (TNF) agents may also reduce the risk of adverse events.

### Tacrolimus

Numerous studies have reported remarkable short‐term therapeutic efficacy in induction of remission therapy of oral tacrolimus (0.1–0.2 mg/kg/day) for steroid‐refractory UC patients and the recommended target trough concentration is 10–15 ng/mL. Furthermore, long‐term therapeutic efficacy and safety of tacrolimus at trough concentrations of 5–10 ng/mL were demonstrated in maintaining remission, so tacrolimus monotherapy may be a potential alternative for those intolerant to AZA.^43^

The principal adverse events of tacrolimus include nephrotoxicity, neurotoxicity, hypertension, hyperglycemia, gastrointestinal disturbances, infections, and malignancy.^44^ Elderly UC patients are more susceptible to various complications.^45^ Especially, the risk of renal dysfunction increases significantly for elderly people.^46^

The experience of tacrolimus therapy in older UC patients is mainly based on case reports. In elderly‐onset refractory UC patients, tacrolimus appears to be effective as remission induction therapy. Notably, tacrolimus serum trough concentrations can rise easily in elderly patients,^43^ hence it is necessary to monitor the drug concentrations and adjust dosage more frequently.

Tacrolimus is metabolized by cytochrome P450 3A enzymes. CYP3A5 gene polymorphism has been proven to affect tacrolimus pharmacokinetics and chronic nephrotoxicity.^47^ CYP3A5 gene polymorphism should be tested to individualize the tacrolimus starting dose and then to optimize the drug exposure.

### Anti‐TNF agents

At present, the data of efficacy of anti‐TNF agents are inconsistent in the treatment of elderly UC patient. In a multicenter study from Italy, the clinical remission rate of elderly UC patients treated with anti‐TNF agents (infliximab or adalimumab) was similar to that of younger patients (59% *vs* 57%, respectively).^5^ However, another study observed lower rate of response to anti‐TNF therapy at 6 months in older IBD patients.^48^ The poor clinical response of elderly patients initiating anti‐TNF treatment is only in the short‐term at 10 weeks, whereas there was no significant difference in the long‐term clinical response at ≥6 months between elderly patients and non‐elderly patients.^49^

However, compared with younger UC patients treated with anti‐TNF agents, elderly patients present higher discontinuation rate of anti‐TNF agents, due to significantly increased risk of infections, malignancy, and possible death.^5^, ^50^ Hence, anti‐TNF agents should be administered to elderly patients with caution.

### Biologic agents targeting leukocyte trafficking

Vedolizumab, as a novel gut‐selective antibody to α4β7 integrin, selectively inhibits inflammation in the gastrointestinal tract.^51^ The short‐term efficacy and long‐term efficacy of vedolizumab for moderate to severe UC were demonstrated in GEMINI 1 and GEMINI 2 studies, with an excellent safety.^52^, ^53^ This gut‐selective mechanism of action may be particularly relevant to elderly UC patients. However, the data of aging patients are limited in size, only 3.7% UC patients were ≥65 years of age in GEMINI studies.^54^ The sparse data demonstrated durable clinical response and clinical remission at week 52 with no noticeable age‐related trends. Older patients treated with vedolizumab had the lowest incidence of serious infections (0.9 per 100 person‐years) and adverse events leading to hospitalization (14.8 per 100 person‐years). Additionally, there were no age‐related differences in the incidence of adverse hematological events, malignancy, or death.

In another study of older patients with IBD treated with vedolizumab, 41.4% achieved clinical remission at 52 weeks with adverse reactions occurring in 13.8%.^55^ Vedolizumab may provide an attractive choice for UC patients, although further investigation of the safety and efficacy of vedolizumab in larger cohorts of elderly patients is needed.

### Usinuzumab

Ustekinumab (UST) is a monoclonal antibody inhibitor of IL‐12/IL‐23 approved for the treatment of CD and UC. A GETAID multicenter real‐world cohort study demonstrated that UST provided steroid‐free clinical remission in one‐third of cases at weeks 12–16 in patients with UC.^56^ Furthermore, the ongoing UNIFI study indicated the long‐term efficacy of UST in patients with UC through 92 weeks. A meta‐analysis of randomized controlled trials indicated that UST did not increase the risk of short‐term adverse events in IBD,^57^ but data in elderly UC patients are lacking.

### Janus kinase inhibitor

Tofacitinib, a small‐molecule Janus kinase (JAK) inhibitor, has recently been approved for the treatment of moderate to severe UC. Tofacitinib inhibits all JAKs, preferentially JAK1 and JAK3, resulting in a wider effect on the gastrointestinal inflammation. Interestingly, it is rapidly absorbed after oral intake with a time to peak concentration of 0.5 h, furthermore the effect of tofacitinib appears to be rapid. The remission rate induced by tofacitinib 10 mg twice daily (BID) at 8 weeks was significantly higher than placebo (16.6% *vs* 3.6%, *P* < 0.001).^58^ Then, high‐certainty evidence also suggests that tofacitinib is superior to placebo for maintenance of remission at 52 weeks in patients with moderate to severe UC.^59^ Tofacitinib seems to have consistent treatment effects in patients who received previous TNF antagonist.^60^ However, the optimal dose of tofacitinib for maintenance therapy is unknown. Tofacitinib has shown an overall good safety profile in UC patients, only herpes zoster infection incidence (5.6%) increased significantly with Tofacitinib.^61^

Unfortunately, data of serious adverse events in elderly patients mainly came from rheumatoid arthritis clinical trials. Serious infections risk was higher in older *versus* younger patients with tofacitinib 10 mg BID but similar between age groups with tofacitinib 5 mg BID and adalimumab, suggesting an effect modification by age for this dose.^62^ An ongoing tofacitinib RA trial in older patients (≥50 years) and with ≥1 cardiovascular risk factor identified a higher frequency of pulmonary embolism (PE) and all‐cause mortality in patients receiving tofacitinib 10 mg BID *versus* those receiving anti‐TNF agent.^63^, ^64^ Currently, few data are available on the risk of venous thromboembolism in UC patients receiving tofacitinib.

Above all, the introduction of tofacitinib adds a new therapeutic strategy for UC patients. Larger studies and longer follow‐up are needed to further address this point.

### Leukocytapheresis

Leukocytapheresis (LCAP) is an extracorporeal therapy for inducing remission of moderate to severe UC. More than 60% UC patients who were treated with LCAP achieved clinical remission within 1 year and remained relapse‐free.^65^ A post hoc analysis of data from Japan demonstrated that the safety and tolerability of LCAP were comparable in the elderly and non‐elderly groups, indicating that it is well tolerated by elderly UC patients.^66^ Interestingly, in another study, the relapse‐free period after LCAP was longer in the older than younger patients.^67^ Thus, LCAP might be an additional effective and safe treatment option for elderly UC patients.

### Colorectal cancer risk and surveillance

Although recent study found that the risk of patients with UC developing colorectal cancer (CRC) has decreased steadily over the last six decades, but the extent and duration of the disease increase this risk. A meta‐analysis based on 116 studies showed that CRC occurred in approximately 3.7% of UC patients, with a cumulative risk of 18% at 30 years.^68^ Incidence rates of CRC were higher in the United States and the United Kingdom compared with other countries. Another study revealed an overall prevalence of CRC of 0.85% among patients with UC in Asia, and cumulative risks of 0.02%, 4.81%, and 13.91% at 10, 20, and 30 years.^69^ In a study based on US population, UC was associated with an increased risk of CRC in elderly patients (odds ratio 1.93; 95% CI 1.54–2.49). More older‐onset UC patients had CRC (3.2% *vs* 0.9%, *P* = 0.033) than non‐older‐onset patients.^8^

On the contrary, recent population‐based studies reported that the incidence of CRC in older‐onset UC patients was similar to that in the general population.^8^, ^70^ And in a multicenter study form United States, there was no difference in the incidence of CRC between older‐onset and adult‐onset UC (3% *vs* 2%; *P* = 0.14).^71^ Nevertheless, older‐onset UC may be related to a more rapid development of CRC.

Therefore, elderly UC patients with longstanding disease require CRC screening sooner after UC diagnosis. Surveillance should be carried out, balancing the age, disease severity, comorbidities, life prognosis, and risk for endoscopy.^12^

## Conclusion

The incidence of UC in elderly patients is increasing. Age‐related problems, including complications, immune dysfunction, and multidrug use, make the diagnosis and treatment of elderly UC more challenging. Thus, it is worth to pay more attention to this special group. Appropriate UC therapy should be selected carefully to balance the clinical outcomes and safety concerns for elderly UC patients.
